# Supplementary material for: Identification of Freezing-Responsive microRNAs and Their Targets in Chinese Jujube by Small RNA and Degradome Sequencing
Source: Int J Mol Sci. 2026 Sep 4;27(17):7912. doi: 10.3390/ijms27177912 (PMC13566890; doi:10.3390/ijms27177912)
Supplement: Supplementary file 1 [file ijms-27-07912-s001.zip › Supplementary Material S2/novel_miR_85.pdf]

Provisional ID : novel_miR_85
Score total : 1.8
Score for star read(s) : -1.3
Score for read counts : 0
Score for mfe : 1.5
Score for randfold : 1.6
Score for cons. seed :
Total read count : 73
Mature read count : 64
Loop read count : 9
Star read count : 0

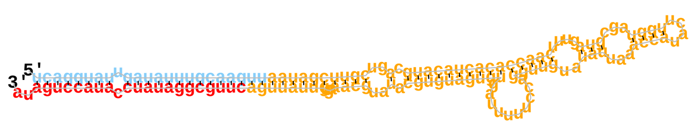

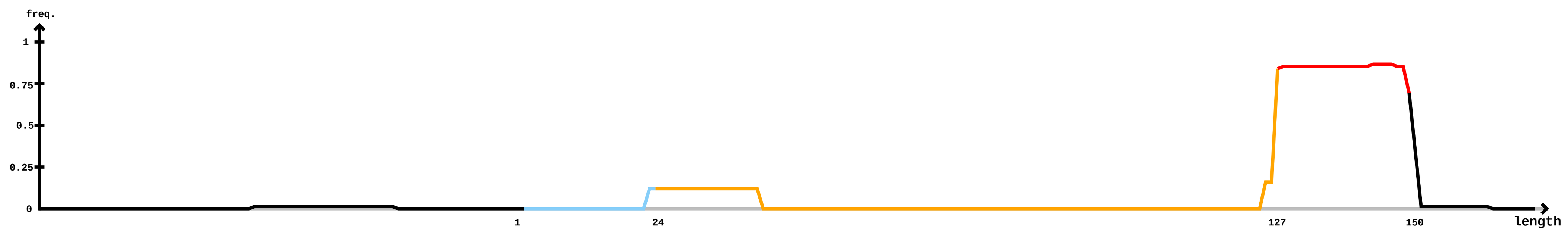

Star Mature

```
5'- ugccaaaaauccaugguuguuucuaguuugaaaaaggcauguuuaggaguuuagaauacauauacauguacaucccuaucagguauugauauuugcaaguuaauagcuugcugacguacaucacaccaacuuugaugcgaugguucauaccaaauuauauguuggaccuuuuuagugugaugugcauaugcaaaguguuauugacuugcggauauccauaccugauaauuauuuuguauauauuucugc -3' exp
    ..((.(((...((((.((.((((......)))).)).))))))).))....(((((...(((((((..........((((((((((.(((((((((((((((((((((((..(.((((((((((((((....(((...((((....))))...)))..)))))..........)))))))))).)..))))...)))))))))))))))))))).))))))))))......)))))))..))))).   reads  mm  sample
    ..................................aggcauguuuaggaguuuagaaua.........................................................................................................................................   1      0   LT-3
    ...........................................................................................uuaauagcuugcugacgua.......................................................................................   9      0   LCK-3
    ...................................................................................................................................................gacuugcggauauccauaccugau.........................   2      0   JCK-1
    ...................................................................................................................................................gacuugcggauauccauaccugau.........................   2      0   JCK-3
    ...................................................................................................................................................gacuugcggauauccauaccuNau.........................   1      1   JCK-1
    ...................................................................................................................................................gacuugcggauauccauaccugau.........................   7      0   JCK-2
    .....................................................................................................................................................cuugcggauauccauaccugaua.......................   22     0   LCK-1
    .....................................................................................................................................................cuugcggauauccauaccugaua.......................   24     0   LCK-2
    .....................................................................................................................................................cuugcggauauccauaccugaua.......................   5      0   LT-2
    ......................................................................................................................................................uugcggauauccauaccug..........................   1      0   JCK-1
    ....................................................................................................................................................................ccugauaauuauuuuguaua.........   1      0   LT-2
```
